# CT‐detected extramural venous invasion‐related gene signature for the overall survival prediction in patients with gastric cancer

**DOI:** 10.1002/cam4.4266

**Published:** 2021-09-12

**Authors:** Bo Gao, Caizhen Feng, Fan Chai, Shengcai Wei, Nan Hong, Yingjiang Ye, Yi Wang, Jin Cheng

**Affiliations:** ^1^ Department of General Surgery Peking University People’s Hospital Beijing China; ^2^ Department of Radiology Peking University People’s Hospital Beijing China; ^3^ Department of Gastrointestinal Surgery Peking University People’s Hospital Beijing China

**Keywords:** extramural venous invasion, gastric cancer, radiogenomics, x‐ray tomography

## Abstract

**Background:**

Computed tomography (CT)‐detected extramural venous invasion (EMVI) has been identified as an independent factor that can be used for risk stratification and prediction of prognosis in patients with gastric cancer (GC). Overall survival (OS) is identified as the most important prognostic indicator for GC patients. However, the molecular mechanism of EMVI development and its potential relationship with OS in GC are not fully understood. In this radiogenomics‐based study, we sought to investigate the molecular mechanism underlying CT‐detected EMVI in patients with GC, and aimed to construct a genomic signature based on EMVI‐related genes with the goal of using this signature to predict the OS.

**Materials and Methods:**

Whole mRNA genome sequencing of frozen tumor samples from 13 locally advanced GC patients was performed to identify EMVI‐related genes. EMVI‐prognostic hub genes were selected based on overlapping EMVI‐related differentially expressed genes and OS‐related genes, using a training cohort of 176 GC patients who were included in The Cancer Genome Atlas database. Another 174 GC patients from this database comprised the external validation cohort. A risk stratification model using a seven‐gene signature was constructed through the use of a least absolute shrinkage and selection operator Cox regression model.

**Results:**

Patients with high risk score showed significantly reduced OS (training cohort, *p* = 1.143e‐04; validation cohort, *p* = 2.429e‐02). Risk score was an independent predictor of OS in multivariate Cox regression analyses (training cohort, HR = 2.758; 95% CI: 1.825–4.169; validation cohort, HR = 2.173; 95% CI: 1.347–3.505; *p* < 0.001 for both). Gene functions/pathways of the seven‐gene signature mainly included cell proliferation, cell adhesion, regulation of metal ion transport, and epithelial to mesenchymal transition.

**Conclusions:**

A CT‐detected EMVI‐related gene model could be used to predict the prognosis in GC patients, potentially providing clinicians with additional information regarding appropriate therapeutic strategy and medical decision‐making.

## Graphical abstract

Imaging feature of computed tomography‐detected extramural venous invasion (EMVI) has been identified as an independent risk factor of gastric cancer (GC). We aimed to investigate the molecular mechanism of EMVI based on whole mRNA genome sequencing of frozen cancerous samples from 13 locally advanced GC. Then using TCGA database, we constructed an EMVI‐related gene model and found it could predict the prognosis in patients with GC.

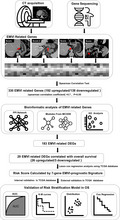

## 1 INTRODUCTION

Gastric cancer (GC) occurs worldwide and is highly heterogeneous. More than 90% of patients with GC in clinical practice have advanced disease, and the radical resection rate is only approximately 50%.^1^ For advanced GC, no specific targeted therapeutic regimen is available, and the prognosis for these patients is poor. The American Joint Committee on Cancer tumor (T), node (N), and metastasis (M) criteria are used for staging GC^2^; in addition, the gross imaging feature extramural venous invasion (EMVI) as detected on computed tomography (CT) has been identified as a promising factor for risk stratification and prediction of prognosis in these patients.^3^, ^4^ Pathologically, EMVI is identified as tumor cells infiltrating through the gastric wall and into the lumen of the extramural vessels,^5^ which can be visualized on CT images. Multiple studies have confirmed that this imaging feature can serve as an independent predictor of synchronous metastasis and progressive event after radial resection in patients with GC.^6^, ^7^ Among multiple prognostic indicators, overall survival (OS) is identified as the definitive and primary end point in cancer clinical trials.^8^

However, in GC, mechanism of EMVI development and its potential relationship with OS are not fully understood. Although CT‐detected EMVI has generally been identified in patients with advanced GC, the prevalence of this imaging feature is <50% in these patients.^6^, ^7^ Furthermore, research has shown that TNM stage is not an influencing factor on the occurrence of blood vessel invasion in GC.^1^ These findings suggest that the development of EMVI is not caused by tumor progression alone. Other research has demonstrated that EMVI detected on pathology may be underestimated when compared with EMVI detected on CT because of sampling difficulties and the destruction of venous endothelial cells in gastrointestinal cancer.^9^ It is therefore necessary to verify the reliability of identifying EMVI on CT and to determine the clinical relevance of this factor in predicting the prognosis in patients with GC.

In recent years, researchers have seen promising results from studies correlating cancer imaging features with high‐throughput data, a research area known as radiogenomics.^10^ Such studies have raised the possibility of increasing precision in diagnosing and predicting prognosis and treatment outcomes in patients with cancer.^11^ For example, a study using The Cancer Genome Atlas (TCGA) data found that the CT imaging feature of acute tumor transition angle was correlated with chromosomal instability in patients with GC.^12^ Radiogenomics could also be used to elucidate the molecular background of these imaging features, which could provide targets for treatment. Regarding EMVI specifically, one study found that TP53 mutations were associated with EMVI on baseline magnetic resonance imaging in patients with rectal cancer,^13^ suggesting that EMVI could be associated with specific molecular (including genomic) characteristics in patients with advanced GC. However, although several studies have assessed use of the genomic signature to predict the prognosis in patients with GC,^14^, ^15^ no studies have addressed the molecular mechanism underlying CT‐detected EMVI based on whole genome sequencing. Because CT‐detected EMVI has been identified as an independent predictor of progression event after surgery in patients with GC, we hypothesized that EMVI‐related genes would be closely related to prognosis.

In this study, we therefore sought to investigate the molecular mechanism underlying CT‐detected EMVI in patients with GC, and we aimed to construct a genomic signature based on EMVI‐related genes with the goal of using this signature to predict the OS.

## 2 MATERIALS AND METHODS

### 2.1 Research strategy

The institutional review board approved this study (approval number: 2019PHB171‐01) and waived the requirement for informed consent because this was a retrospective analysis.

**FIGURE 1:**
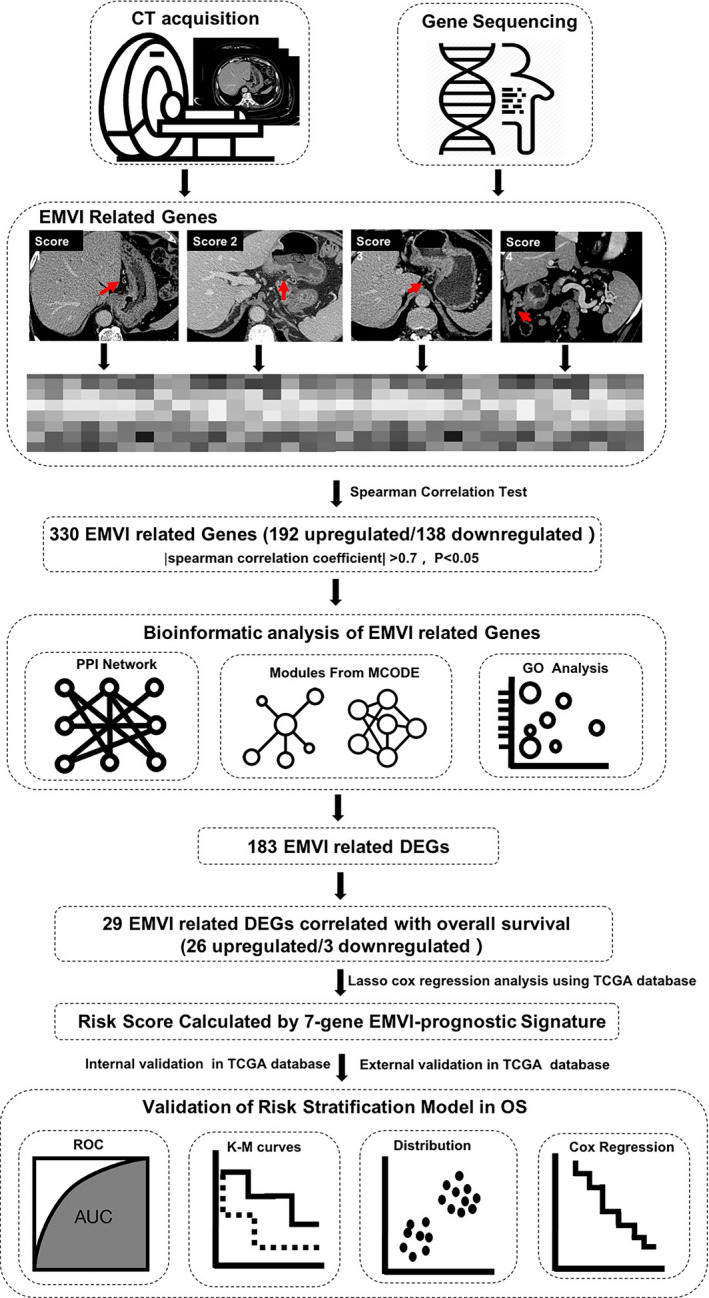
Flowchart of the study. AUC, area under the curve; CT, computed tomography; DEGs, differentially expressed genes; EMVI, extramural venous invasion; GO, gene ontology; K–M, Kaplan–Meier; OS, overall survival; PPI, protein–protein interaction; ROC, receiver operating characteristic; TCGA, The Cancer Genome Atlas

The flowchart of the study is shown in Figure 1. First, tumor samples were collected from patients with GC with various EMVI scores, and gene sequencing was performed. Second, EMVI‐related genes were identified based on EMVI score and gene expression data. Third, bioinformatic analyses were performed for EMVI‐related genes, including protein–protein interaction (PPI) network establishment, gene module construction in the network, and gene ontology (GO) analyses. Fourth, EMVI‐related differentially expressed genes (DEGs) and OS‐related DEGs were identified through univariate and least absolute shrinkage and selection operator (LASSO) Cox regression analyses. Fifth, an EMVI‐prognostic gene model was proposed using the TCGA database for training and external validation cohorts. Sixth, GO and Kyoto Encyclopedia of Genes and Genomes (KEGG) analyses of the EMVI‐prognostic gene model were performed.

### 2.2 Patient selection

A search of the hospital's histopathologic electronic information system was performed, and 13 patients with locally advanced GC who underwent contrast‐enhanced multidetector CT (ceMDCT) followed by standard D2 radical gastrectomy between January 2018 and January 2019 were identified and included in the study. Included patients also received adjuvant chemotherapy using the SOX regimen (Oxaliplatin 130 mg/m^2^ IV on day 1 plus S‐1 40–60 mg [<1.25 m^2^, 40 mg; 1.25–1.5 m^2^, 50 mg; and >1.5 m^2^, 60 mg] twice daily PO for 14 days, repeat every 3 weeks). Follow‐up included laboratory tests (whole blood count, carcinoembryonic antigen) and chest/abdominal ceMDCT scans every 2–3 months within 1 year and then about every 6 months postoperatively. Data regarding event of tumor recurrence, metastasis, and death after surgery as well as OS time were recorded.

Exclusion criteria included: (1) patients who had radiology and/or pathology confirmed metastasis; (2) patients who had been given neoadjuvant treatment before surgery, or treated with palliative surgery; (3) patients who underwent MDCT without contrast agent; and (4) patients who had a history of another malignant tumor.

### 2.3 MDCT image acquisition and EMVI detection

Abdominal ceMDCT scans (covering from the top of the diaphragm to the symphysis pubis) were performed using a 256‐MDCT scanner (Brilliance iCT; Philips Healthcare). After fasting for 8 h, patients ingested 600–800 ml of water 5 min before image acquisition to distend the stomach. A power injector (Missouri XD2001; Ulrich) was then used to administer intravenous iodinated contrast agent (80 ml iopromide, 370 mg/ml; Ultravist, Bayer Schering Pharma) through the antecubital vein at a rate of 2.5 ml/s. Images were acquired using the following parameters: 120 kV, 240–400 mA, and 5‐mm slice thickness and increments. The late arterial and portal venous CT phases were initiated at 10 and 45 s, respectively, after the trigger threshold (100 Hounsfield units on the abdominal aorta) had been reached. Axial, sagittal, and coronal reconstructions with 1.25‐mm thickness were then performed on a dedicated workstation (Advantage Workstation 4.3; GE Healthcare).

CT‐detected EMVI status was reviewed on the preoperative ceMDCT images. EMVI scores were defined as follows^16^, ^17^: score 0, pattern of tumor extension through the gastric wall is not nodular, and without vessels adjacent to tumor penetration area; score 1, nodular tumor extension but no vessels in the area; score 2, stranding demonstrated in the vicinity of normal extramural vessels; score 3, tubular soft tissue extends from the tumor, resulting in filling defect within extramural vessels; and score 4, nodular soft tissue extends from the tumor, resulting in obvious irregular vessel contour. In this system, scores ranging from 0 to 2 are considered EMVI negative, whereas scores of 3 and 4 are considered EMVI positive.

### 2.4 Gene sequencing and identification of EMVI‐related genes in GC

Fresh‐frozen tumor tissue samples from the surgical resections (obtained before chemotherapy treatment) were collected from the institutional biobank. After quality control tests were performed, the Illumina NextSeq system was used to sequence the whole mRNA expression of these samples.

Based on these sequencing data, we used R language to calculate the Spearman correlation between gene expression and EMVI. The threshold of correlation significance was set at |Spearman correlation coefficient| >0.7 and *p* < 0.05. The R language heatmap package was used to draw the heatmap of EMVI‐related genes in GC.

### 2.5 PPI network, module construction, and GO analyses of EMVI‐related genes

The STRING database (version 11.0) (http://string‐db.org/) and Cytoscape software (3.7.1 version) were used to establish the PPI network of EMVI‐related genes in GC. MCODE, a Cytoscape plugin that finds highly interconnected regions in a network, was used to identify the EMVI‐related gene modules in the PPI network.^18^ The GO categories for the selected modules were derived from the Database for Annotation, Visualization, and Integrated Discovery (DAVID; http://david.ncifcrf.gov). A hypergeometric distribution test was used to identify significant GO terms (*p* < 0.01).

### 2.6 Hub gene selection based on overlapping EMVI‐related DEGs and OS‐related genes

Among the EMVI‐related genes, we identified DEGs according to EMVI status (positive vs. negative) using the R limma package, with a threshold of *p* < 0.05. We then downloaded mRNA sequencing data and corresponding clinical information from 350 patients with GC included on the TCGA database using official download tool Genomic Data Commons (http://portal.dgc.cancer.gov; data through 24 October 2020, used). TCGA database is a cancer research project jointly established by the National Cancer Institute and the National Human Genome Institute. The database includes more than 20,000 primary cancer and matched normal samples spanning 33 cancer types. The cases from the TCGA database were divided randomly into training (*n* = 176) and external validation (*n* = 174) cohorts. OS‐related genes in the training cohort were selected using the R limma package, with a threshold of *p* < 0.05. EMVI‐prognostic hub genes were then selected based on overlapping EMVI‐related DEGs and OS‐related genes. A univariate Cox analysis of OS was performed to determine the prognostic value of these hub genes. An interaction network for hub genes was generated using the STRING database (version 11.0).

### 2.7 Construction and validation of EMVI‐prognosis‐related gene model

For construction of the EMVI‐prognosis‐related gene signature, the LASSO algorithm was used for variable selection and shrinkage with the “glmnet” R package. The independent variable in the regression was the normalized expression matrix of candidate EMVI‐prognosis‐related genes, and the response variables were OS and status of patients in the training cohort of the TCGA database. The penalty parameter (λ) for the model was determined using 10‐fold cross‐validation following the minimum criteria. The risk value of the risk‐related EMVI‐related hub genes was calculated using the following formula:

$$\text{Risk score}=\sum_{i=1}^{n}\text{Coefi}\times xi,$$

where Coefi refers to the regression coefficient and *xi* refers to the *z*‐score–transformed relative expression value. By multiplying the coefficient and the expression of EMVI‐prognosis‐related hub genes, we were able to obtain the risk value of each gene. The patients were stratified into high‐ and low‐risk groups based on the median value of the risk score. Principle component analysis (PCA) and t‐distributed stochastic neighbor embedding (t‐SNE) were performed using the stats and Rtsne R packages, respectively. Kaplan–Meier curves were calculated, and log‐rank tests and receiver operating curve (ROC) analyses were also performed. Independent prognostic parameters analysis was performed using univariate and multivariate Cox regression analyses. The candidate parameters included age, sex, tumor differentiation grade, disease stage, and risk score were calculated using the EMVI‐prognosis‐related gene signature.

### 2.8 Functional and KEGG enrichment analyses of EMVI‐prognosis‐related gene model

The R clusterProfiler package was used to conduct GO and KEGG analyses based on DEGs in the EMVI‐prognosis‐related model.

## 3 RESULTS

### 3.1 Clinical characteristics

The 13 study patients (3 women and 10 men) had a mean age of 71.54 years (range, 60–83 years). All patients had histopathologically proven T4aN+M0 disease based on a surgical specimen. All patients had GC in the distal part of the stomach, with all tumor types identified as low differential adenocarcinoma, intestinal classification. Six patients were EMVI positive, and the remaining seven patients were EMVI negative. Four patients had a progression event within 1 year after surgery; three of these patients were EMVI positive. The median follow‐up time was 20 months (interquartile range: 13.5 months) (Table 1).

**TABLE 1:** Patient characteristics

No.	Sex	Age (years)	EMVI score	pT	pN	Recurrence/metastasis	Death	OS (months)
1	F	82	1	T4a	N1	No	Yes	18
2	M	65	2	T4a	N2	No	No	35
3	F	77	2	T4a	N1	No	No	30
4	M	77	4	T4a	N1	Liver metastases	Yes	12
5	M	73	3	T4a	N3b	No	No	28
6	M	69	4	T4a	N2	Local recurrence	Yes	12
7	M	72	4	T4a	N1	No	No	24
8	M	81	3	T4a	N3b	Peritoneal metastases	Yes	10
9	M	60	2	T4a	N3a	No	No	22
10	M	67	2	T4a	N3a	No	No	20
11	M	83	1	T4a	N3a	Peritoneal metastases	Yes	20
12	M	64	2	T4a	N2	Liver metastases	Yes	12
13	M	60	3	T4a	N2	No	No	18

Abbreviations: EMVI, extramural venous invasion; OS, overall survival.

### 3.2 Identification of related genes based on CT‐detected EMVI

**FIGURE 2:**
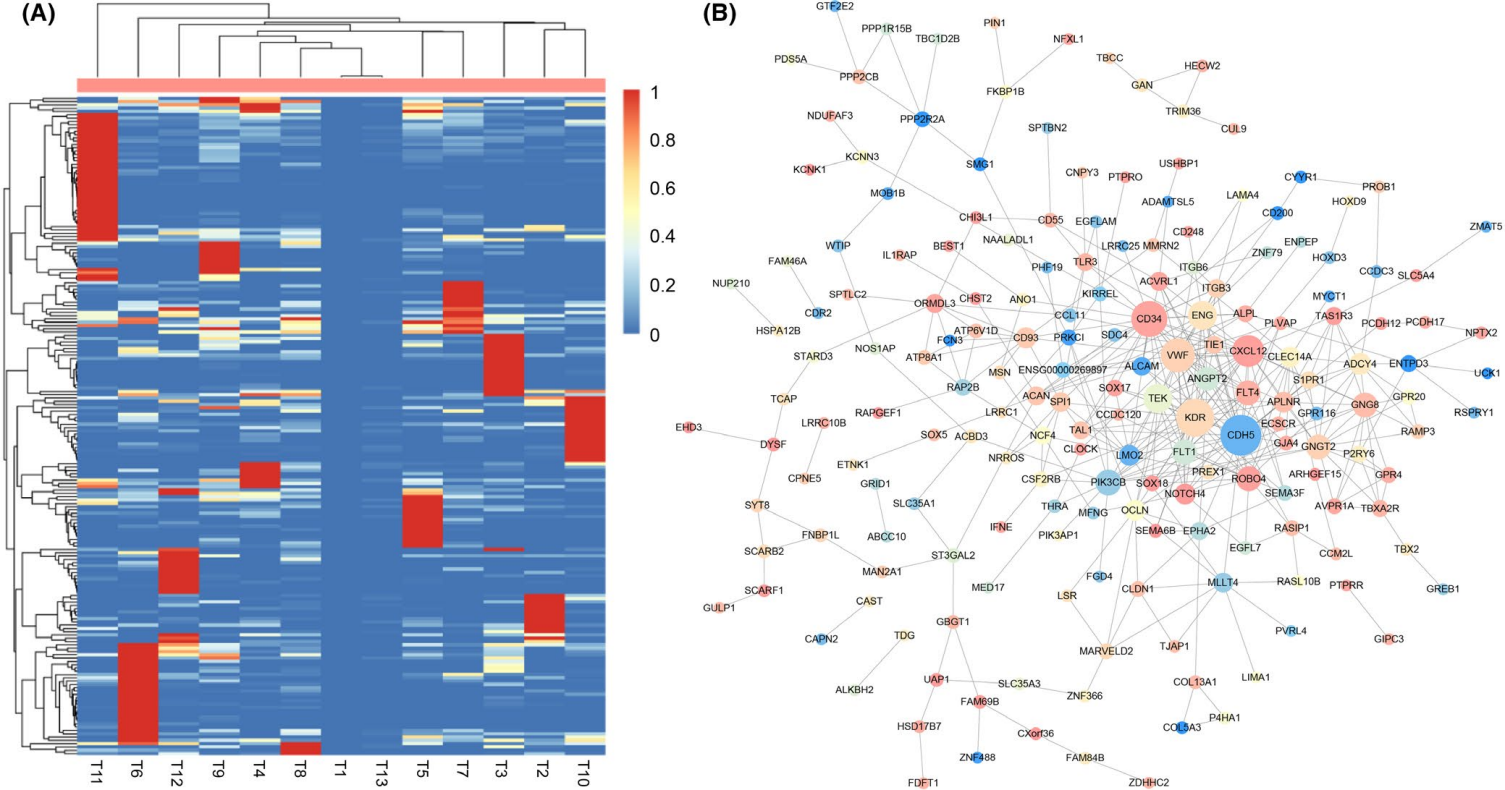
The heatmap (A) and protein–protein interaction network (B) of extramural venous invasion (EMVI)‐related genes in gastric cancer. In (B), the color of the node represents the *p* value of the correlation between EMVI and gene expression

A total of 330 EMVI‐related genes were identified, including 192 EMVI‐positive correlation genes and 138 EMVI‐negative correlation genes. A heatmap of these EMVI‐related genes is shown in Figure 2A.

### 3.3 PPI network, module construction, and GO analyses of EMVI‐related genes

**FIGURE 3:**
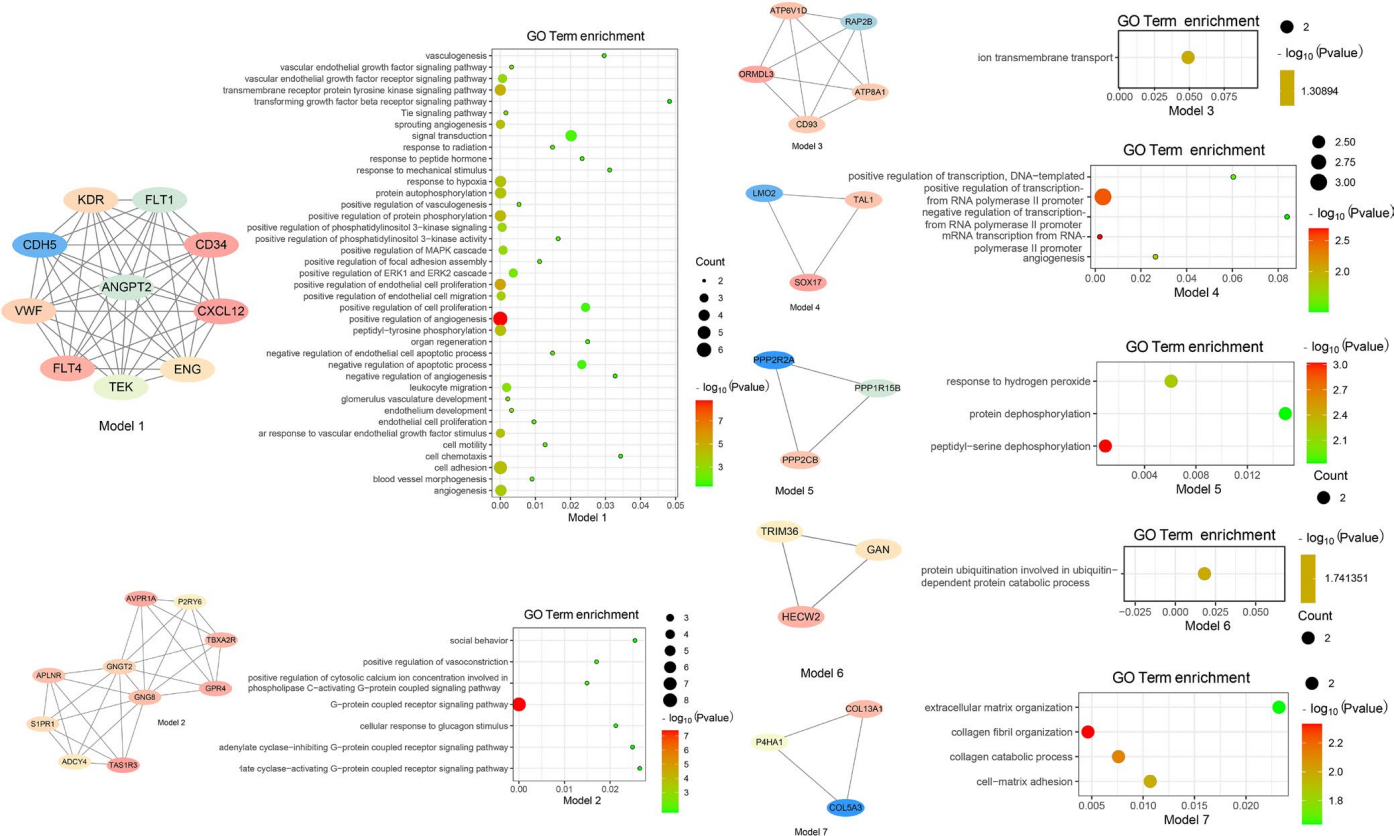
Gene ontology (GO) analysis of modules

We identified 395 PPI pairs among the 330 EMVI‐related genes and established a PPI network (Figure 2B). Seven modules were selected as highly interconnected regions in the PPI network (Figure 3). GO terms with a *p* value cutoff of 0.01 were primarily involved in angiogenesis, G protein‐coupled receptor signaling pathways, cell proliferation, cell adhesion, and ion transmembrane transport.

### 3.4 Hub gene selection based on overlapping EMVI‐related DEGs and OS‐related genes

**FIGURE 4:**
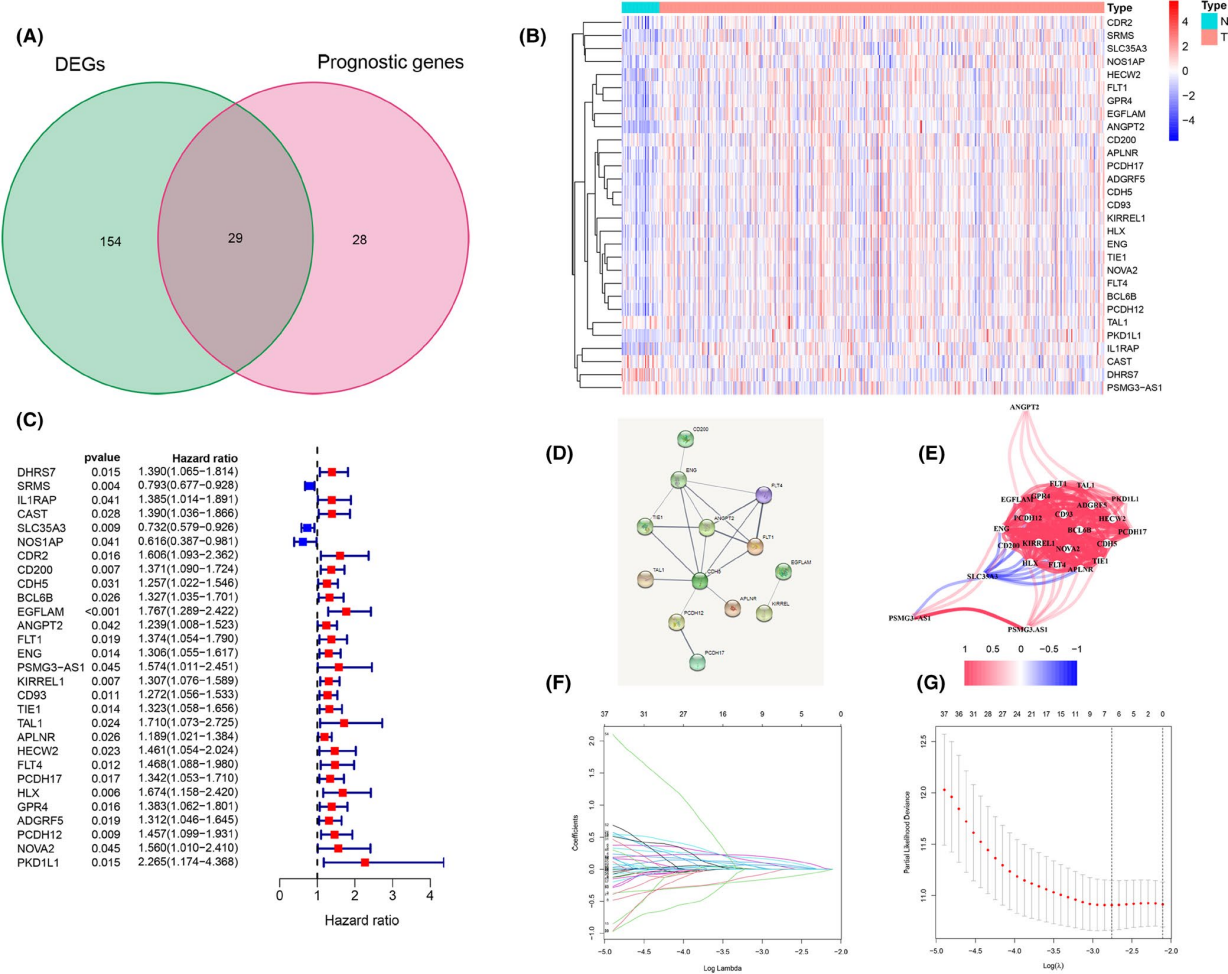
Identification of extramural venous invasion (EMVI)‐prognosis‐related differentially expressed genes (DEGs) in The Cancer Genome Atlas cohort. (A) Venn diagram used to identify EMVI‐related DEGs (EMVI positive vs. EMVI negative) that were correlated with overall survival (OS). (B) A total of 26 overlapping genes were upregulated and 3 were downregulated. (C) Forest plots showing the results of the univariate analysis for gene expression and OS. (D) The protein–protein interaction network indicates interactions among the candidate DEGs. (E) The correlation network of candidate DEGs. The correlation coefficients are represented with different colors. (F) The coefficients were changed with the change of λ through use of the “glmnet” package in R language. (G) The best lambda λ was found using 10‐fold cross‐validation

A total of 183 DEGs were identified. Among these, a total of 29 DEGs (26 upregulated and three downregulated) overlapped with OS‐related genes (57 genes) in the training cohort (Figure 4A). The heatmap of these 29 genes is shown in Figure 4B, and the Forest diagram is shown in Figure 4C. The interaction network among these genes indicated that ANGPT2 and COH5 were the hub genes (Figure 4D). The correlation between these genes is shown in Figure 4E.

### 3.5 Construction of EMVI‐prognosis‐related gene model in TCGA cohort

**FIGURE 5:**
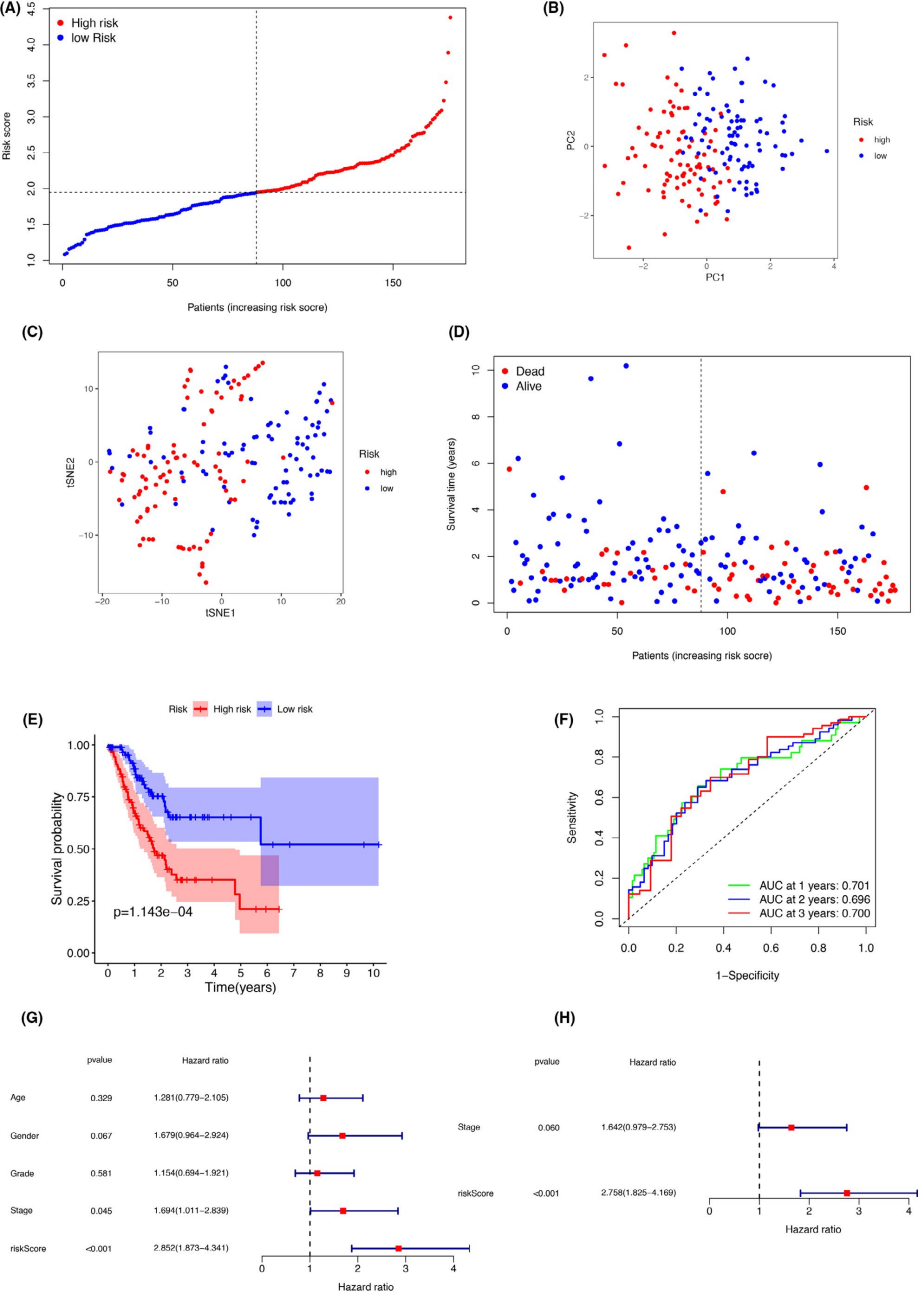
Prognostic analysis of the seven extramural venous invasion‐prognosis‐related differentially expressed genes (DEGs) in the training cohort. (A) Distribution and median value of risk scores. (B) Principle component analysis and (C) t‐distributed stochastic neighbor embedding analysis plot showing patients in different risk groups were distributed in two directions. (D) The distribution of overall survival (OS) and risk score. (E) Kaplan–Meier curves for the OS of patients in high‐risk and low‐risk groups. (F) Area under the curve (AUC) for time‐dependent receiver operating characteristic curves demonstrating the prognostic performance of the risk score. (G) Univariate Cox regression analysis showing that age, gender, tumor grade, tumor stage, and risk score were significant factors of OS in GC patients. (H) Multivariate Cox regression analysis showing that disease stage and risk score were the independent factors for the OS in GC patients. GC, gastric cancer

LASSO Cox regression analysis was applied to establish a prognostic model using the expression profile of the 29 hub genes mentioned earlier. A seven‐gene signature (SRMS, GULP1, CAST, NOS1AP, ERRFI1, ENPEP, and EHD3) was identified based on the optimal value of λ (Figure 4F,G). The training cohort cases were stratified into a high‐risk group (*n* = 88) and a low‐risk group (*n* = 88) according to the median cutoff value (high‐risk group: risk score ≥1.95; low‐risk group: risk score <1.95) (Figure 5A, Table 2). PCA and t‐SNE analyses demonstrated that the patients in different risk groups were distributed in two directions (Figure 5B,C). Patients in the high‐risk group had a higher probability of earlier death than those in the low‐risk group (Figure 5D). Kaplan–Meier curve analysis demonstrated that patients in the high‐risk group had a significantly lower OS than those in the low‐risk group (*p* = 1.143e‐04) (Figure 5E). The area under the curve (AUC) values for the ability of the risk score to predict the OS were 0.701 at 1 year, 0.696 at 2 years, and 0.700 at 3 years (Figure 5F). Univariate and multivariate Cox regression analyses demonstrated that tumor stage (HR = 1.642; 95% CI: 0.979–2.753) and risk score (HR = 2.758; 95% CI: 1.825–4.169) were independent predictors of OS (Figure 5G,H).

**TABLE 2:** Baseline characteristics of patients in the training cohort included for univariate and multivariate Cox regression analyses

Clinical feature	All patients (*n* = 176)	High‐risk group (*n* = 88)	Low‐risk group (*n* = 88)
	*n* (%)	*n* (%)	*n* (%)
Age (years)			
≤65	81 (46.0)	42 (47.7)	39 (44.3)
>65	95 (54.0)	46 (52.3)	49 (55.7)
Sex			
Female	59 (33.5)	28 (31.8)	31 (35.2)
Male	117 (66.5)	60 (68.2)	57 (64.8)
Grade			
1–2	69 (39.0)	27 (30.7)	42 (47.7)
3–4	106 (60.0)	61 (69.3)	45 (51.2)
Unknown	1 (1.0)	0 (0)	1 (1.1)
Stage			
I–II	83 (47.2)	37 (42.1)	46 (52.3)
III–IV	87 (49.4)	48 (54.5)	39 (44.3)
Unknown	6 (3.4)	3 (3.4)	3 (3.4)
Stage T			
1–2	48 (27.3)	22 (25.0)	26 (29.5)
3–4	126 (71.6)	65 (73.9)	59 (67.1)
Unknown	2 (1.1)	1 (1.1)	3 (3.4)
Stage M			
M0	163 (92.6)	80 (90.9)	83 (94.3)
M1	11 (6.3)	7 (8.0)	4 (4.6)
Unknown	2 (1.1)	1 (1.1)	1 (1.1)
Stage N			
N0	46 (26.0)	17 (19.3)	29 (33.0)
N1–3	123 (70.0)	68 (77.3)	55 (62.5)
Unknown	7 (4.0)	3 (3.4)	4 (4.5)

### 3.6 External validation of the EMVI‐prognosis‐related gene model in TCGA cohort

**FIGURE 6:**
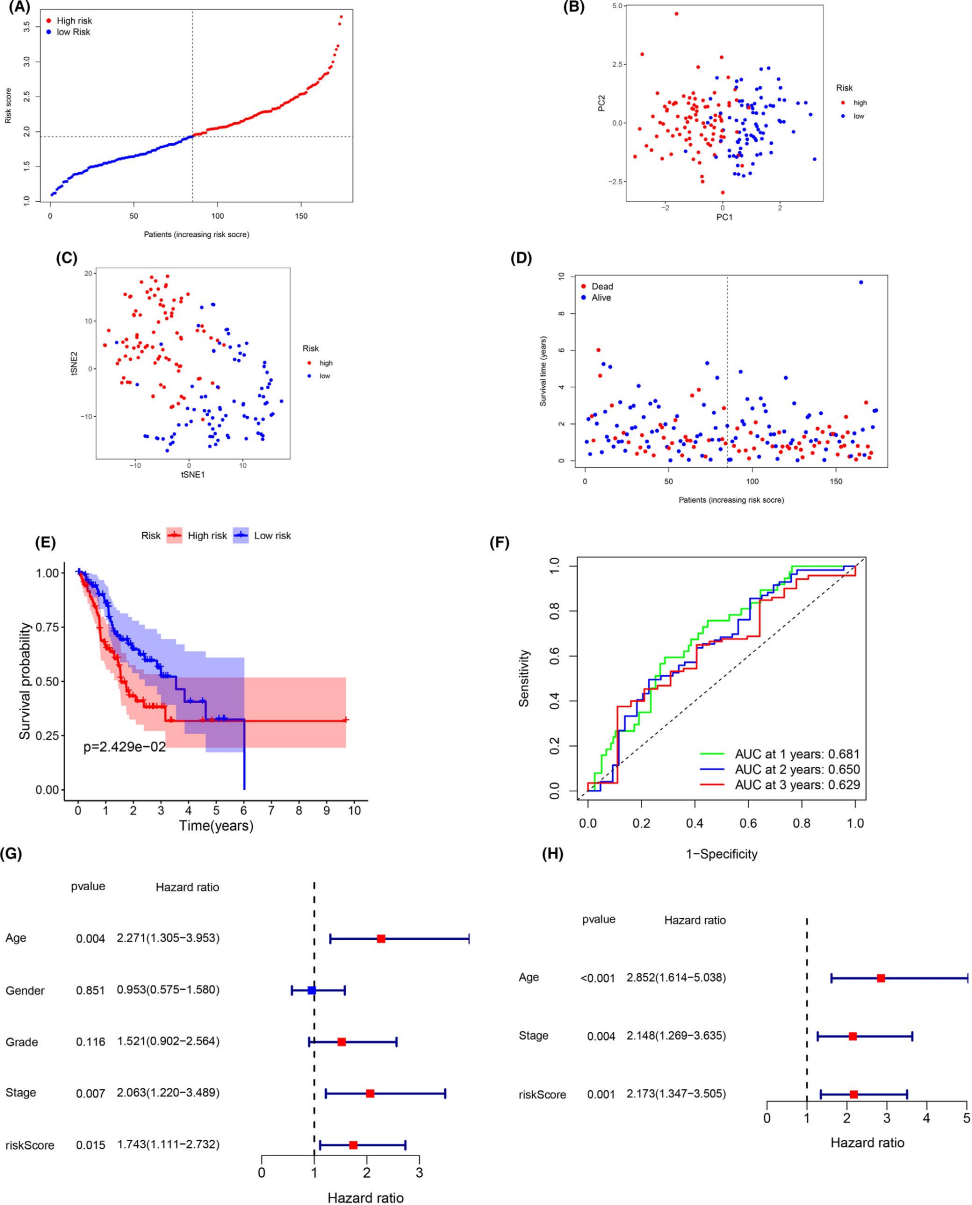
Prognostic validation of the seven extramural venous invasion‐prognosis‐related differentially expressed genes (DEGs) in the external validation cohort. (A) Distribution and median value of risk scores. (B) Principle component analysis and (C) t‐distributed stochastic neighbor embedding analysis showing patients in different risk groups were distributed in two directions. (D) The distribution of overall survival (OS) and risk score. (E) Kaplan–Meier curves for the OS of patients in high‐risk group and low‐risk groups. (F) Area under the curve (AUC) for time‐dependent receiver operating characteristic curves demonstrating the prognostic performance of the risk score. (G) Univariate Cox regression analysis showing that age, gender, tumor grade, tumor stage, and risk score were significant factors of OS in GC patients. (H) Multivariate Cox regression analysis showing that age, stage, and risk score were the independent factors for the OS in GC patients. GC, gastric cancer

Patients from the external validation cohort were similarly divided into a high‐risk group (*n* = 89) and a low‐risk group (*n* = 85) (Figure 6A, Table 3). As in the training cohort, the high‐risk and low‐risk groups in this cohort demonstrated different distributions in PCA, t‐SNE, and OS (Figure 6B–D). Kaplan–Meier and ROC analyses demonstrated that the patients in the high‐risk group had a lower OS than those in the low‐risk group (Figure 6E). The AUC values were 0.681 at 1 year, 0.650 at 2 years, and 0.629 at 3 years (Figure 6F). Univariate and multivariate regression analyses identified age (HR = 2.852; 95% CI: 1.641–5.038), tumor stage (HR = 2.148; 95% CI: 1.262–3.635), and risk score (HR = 2.173; 95% CI: 0.1.347–3.505) as independent prognostic predictors of OS (Figure 6G,H).

**TABLE 3:** Baseline characteristics of patients in the external validation cohort included for univariate and multivariate Cox regression analyses

Clinical feature	All patients (*n* = 174)	High‐risk group (*n* = 89)	Low‐risk group (*n* = 85)
	*n* (%)	*n* (%)	*n* (%)
Age (years)			
≤65	77 (44.3)	41 (46.1)	36 (42.4)
>65	97 (55.7)	48 (53.9)	49 (57.6)
Sex			
Female	65 (37.4)	33 (37.1)	32 (37.6)
Male	109 (62.6)	56 (62.9)	53 (62.4)
Grade			
1–2	65 (37.4)	27 (30.3)	38 (44.7)
3–4	101 (58.0)	56 (62.9)	45 (52.9)
Unknown	8 (4.6)	6 (6.8)	2 (2.4)
Stage			
I–II	73 (42.0)	35 (39.3)	38 (44.7)
III–IV	93 (53.4)	50 (56.2)	43 (50.6)
Unknown	8 (4.6)	4 (4.5)	4 (4.7)
Stage T			
1–2	42 (24.1)	17 (19.1)	25 (29.4)
3–4	130 (74.7)	70 (78.7)	60 (70.6)
Unknown	2 (1.1)	2 (2.2)	0 (0)
Stage M			
M0	149 (85.6)	76 (85.4)	73 (85.6)
M1	12 (6.9)	7 (7.9)	5 (5.9)
Unknown	13 (7.5)	6 (6.7)	7 (8.2)
Stage N			
N0	57 (32.8)	27 (30.3)	30 (35.3)
N1–3	113 (64.9)	59 (66.3)	54 (63.5)
Unknown	4 (2.3)	3 (3.4)	1 (1.2)

### 3.7 Functional and KEGG enrichment analyses of EMVI‐prognosis‐related gene model

**FIGURE 7:**
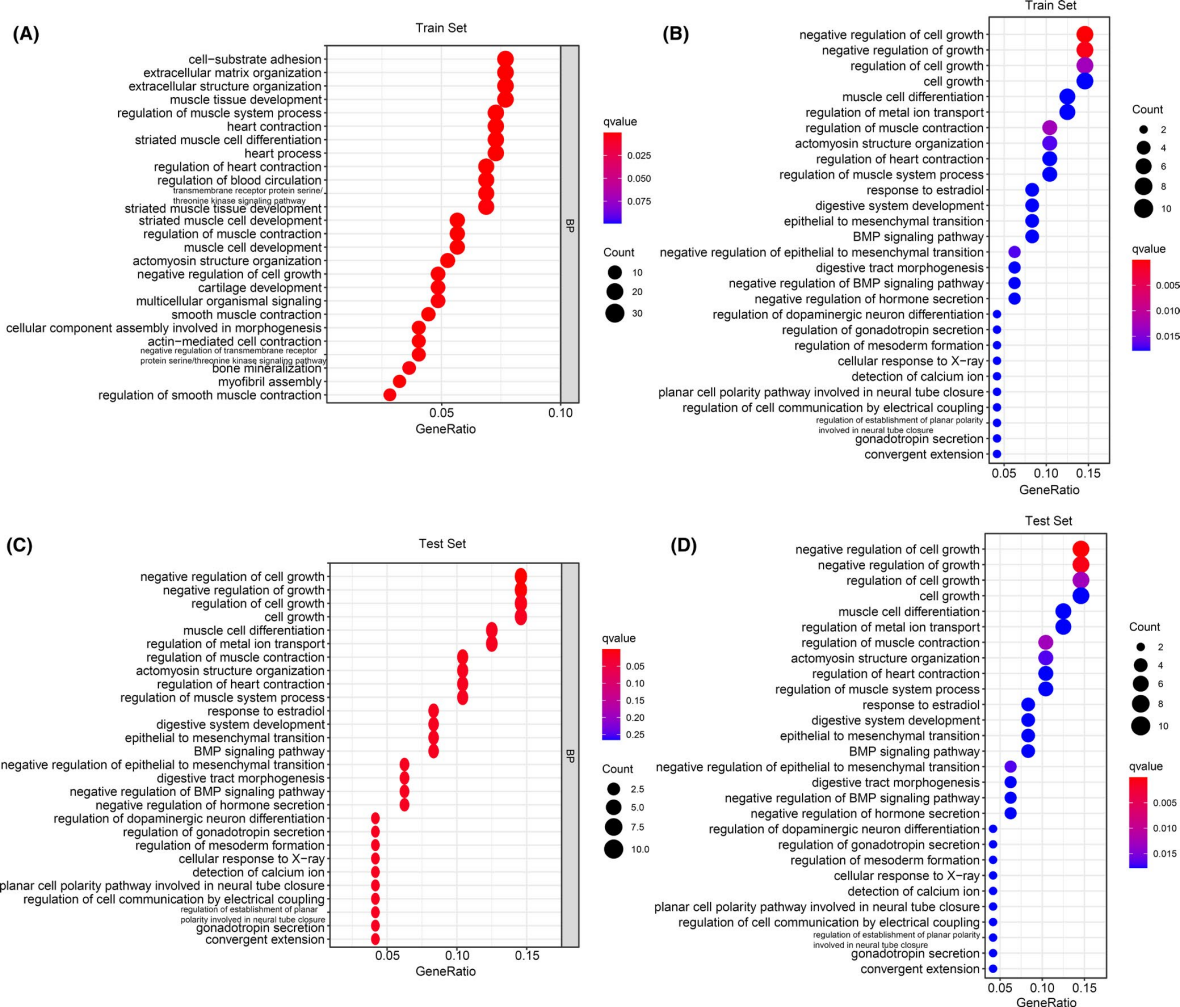
Gene ontology (A, C) and Kyoto Encyclopedia of Genes and Genomes (B, D) analyses of seven genes in the extramural venous invasion‐prognosis‐related model in the training (A, B) and external validation (C, D) cohorts

GO and KEGG analyses performed for the training and external validation cohorts, respectively, demonstrated that the genomic functions and pathways were enriched in cell growth, cell adhesion, regulation of metal ion transport, extracellular organization, and epithelial to mesenchymal transition (Figure 7).

## 4 DISCUSSION

In this radiogenomics‐based study, we investigated the molecular background of CT‐detected EMVI in patients with GC, and we found that EMVI‐related genes were enriched by multiple oncology‐related functions. We then established a seven‐gene model derived from EMVI‐prognosis‐related genes that could be used to predict the prognosis in this patient population.

The gross imaging feature of CT‐detected EMVI has been identified as an important independent predictor of poor prognosis in patients with GC,^4^, ^6^, ^19^ and it has also been found to be associated with tumor burden, lymph node metastasis, and distant metastasis.^7^, ^19^ However, EMVI in patients with GC is difficult to accurately diagnose on pathologic analysis.^16^ We sought to investigate the molecular background of the development of CT‐detected EMVI in patients with GC, and our bioinformatic analysis demonstrated that multiple oncologic‐related functions were enriched in EMVI‐related genes, including angiogenesis, G protein‐coupled receptor signaling pathways, RNA polymerase II promoter, collagen fibril organization, and ion transmembrane transport. These findings are consistent with the clinical effect of EMVI on tumor proliferation and metastasis.

Previous research has shown that vascular endothelial growth factor (VEGF) induces endothelial and cancer cell migration^20^ and is associated with tumor growth, transmural extension, local lymphatic metastases, and distant metastasis of malignant tumor in patients with gastrointestinal cancer.^21^ Additionally, correlations have been found between EMVI scores on magnetic resonance imaging and VEGF expression in T3 rectal cancers.^22^ Taken together, these findings suggest that the process of angiogenesis could be a precondition for the development of EMVI. mRNA expression of genes involved in vascular morphogenesis and early vessel mutation may therefore be a promising predictor of response to anti‐angiogenetic chemotherapy such as ramucirumab, a selective VEGFR2 monoclonal antibody that has been found to improve the clinical outcomes in patients with advanced disease.^23^, ^24^

Other EMVI‐related oncological genes are also related to the function of angiogenesis. G protein‐coupled receptor, a promising oncogene related to tumor cell proliferation and migration in GC,^25^ has been verified to be a controller of a number of angiogenic signals.^26^ RNA polymerase II promoter has been identified as an enhancer of the VEGFA pathway.^27^ Cortistatin A, one of the inhibitors of transcription‐associated cyclin‐dependent kinase, may reduce VEGF‐induced migration.^28^ Additionally, in terms of ion transmembrane transport, the process of vascular network remodeling may be associated with fluxes of ions and other small molecules mediated by the ion channels and transporters.^29^

Of the EMVI‐related genes identified in this study, 29 DEGs overlapped between CT‐detected EMVI genes and OS‐related DEGs. The EMVI‐prognostic model constructed using LASSO Cox regression in this study was able to predict the OS in both the training and external validation cohorts, with acceptable AUC values. Furthermore, the risk score calculated using this gene model was found to be an independent predictor of OS in patients with GC. Multiple genomic functions/pathways were enriched in EMVI‐related genes not only in the training cohort, but also in the external validation cohort. These genomic functions/pathways were all closely related to tumor proliferation and metastasis, potentially explaining the ability of this model to predict the OS. However, angiogenesis was not identified as an enriched function of EMVI‐prognosis‐related genes, suggesting that prognosis is influenced by multiple genomic mediations.

The prognostic model proposed in this study includes seven EMVI‐related DEGs: SRMS, GULP1, CAST, NOS1AP, ERRFI1, ENPEP, and EHD3. Previous research has demonstrated that Src‐family kinase‐mediated phosphorylation of cellular substrates plays an important role in mitosis, cell spread, adhesion, motility, cell death, survival, and differentiation.^30^ As a member of the Src family, SRMS has been found to be critical in epidermal growth factor (EGF)‐stimulated phosphorylation of Sam 68, a major RNA‐binding protein.^31^ In breast cancer, the levels of SRMS expression have been found to be correlated with the grade and severity of the tumor.^32^ Similarly, NOSIAP has been found to promote tumor cell migration in breast cancer.^33^ CAST, a metal ion transition gene, and corresponding proteins such as calpain have been identified as positive factors in tumorigenesis and tumor progression in GC.^34^ ENPEP is known to be associated with inflammatory or immune responses that may be associated with the mechanisms of depressive disorder.^35^ ENPEP has also been identified as one of the genes involved in the four‐gene model for the prediction of prognosis in colorectal cancer.^36^ The remaining three genes in our seven‐gene model are tumor‐suppressing genes. ERRFI1 inhibits growth and enhances response to chemotherapy in cells expressing high levels of EGF receptor (EGFR).^37^ EHD3 has been found to be correlated with the EGFR signaling pathway, potentially explaining the higher sensitivity of EHD3‐expressing cells to the growth‐inhibitory effects of EGF.^38^ Finally, GULP1 (PTB domain‐containing engulfment adaptor protein 1) has been found to be inactivated in ovarian cancer by promoter methylation, which is inversely correlated with expression.^39^

This study had several limitations. First, the study had a small sample size for CT‐detected EMVI‐related gene selection. Second, the main source of our clinical information was a dataset from the TCGA database, and most of these patients were White, African, or Latino; however, the model we created did show moderate predictive ability in the external validation cohort despite this limitation. Third, the protein expression levels associated with the molecular mechanisms of EMVI development require further study.

In conclusion, this study demonstrated that CT‐detected EMVI‐related genes are enriched by multiple oncology‐related functions. The CT‐detected EMVI‐related gene model constructed in this study could be used to predict the prognosis in patients with GC, which could assist clinicians with therapeutic decision‐making.

## CONFLICT OF INTEREST

The authors declare that they have no conflict of interest.

## ETHICAL APPROVAL

This study was approved by the institutional review board under Approval number: 2019PHB171‐01, which waived the requirement for obtaining informed consent.

## Data Availability

The data used to support the findings of this study are available from the corresponding author upon request.
